# Gestalt assessment of online educational resources may not be sufficiently reliable and consistent

**DOI:** 10.1007/s40037-017-0343-3

**Published:** 2017-02-27

**Authors:** Keeth Krishnan, Brent Thoma, N. Seth Trueger, Michelle Lin, Teresa M. Chan

**Affiliations:** 1 University of Toronto, Toronto, Ontario Canada; 2 Department of Emergency Medicine, University of Saskatchewan, Saskatoon, Saskatchewan Canada; 3 Department of Emergency Medicine, Northwestern University, Chicago, IL USA; 4 Department of Emergency Medicine, University of California San Francisco, San Francisco, CA USA; 5 Division of Emergency Medicine, Department of Medicine, McMaster University, Hamilton, Ontario Canada

**Keywords:** Critical appraisal, E-learning, Free open access medical education (FOAM)

## Abstract

**Purpose:**

Online open educational resources are increasingly used in medical education, particularly blogs and podcasts. However, it is unclear whether these resources can be adequately appraised by end-users. Our goal was to determine whether gestalt-based recommendations are sufficient for emergency medicine trainees and attending physicians to reliably recommend online educational resources to others.

**Methods:**

Raters (33 trainees and 21 attendings in emergency medicine from North America) were asked to rate 40 blog posts according to whether, based on their gestalt, they would recommend the resource to (1) a trainee or (2) an attending physician. The ratings’ reliability was assessed using intraclass correlation coefficients (ICC). Associations between groups’ mean scores were assessed using Pearson’s *r*. A repeated measures analysis of variance (RM-ANOVA) was completed to determine the effect of the level of training on gestalt recommendation scale (i. e. trainee vs. attending).

**Results:**

Trainees demonstrated poor reliability when recommending resources for other trainees (ICC = 0.21, 95% CI 0.13–0.39) and attendings (ICC = 0.16, 95% CI = 0.09–0.30). Similarly, attendings had poor reliability when recommending resources for trainees (ICC = 0.27, 95% CI 0.18–0.41) and other attendings (ICC = 0.22, 95% CI 0.14–0.35). There were moderate correlations between the mean scores for each blog post when either trainees or attendings considered the same target audience. The RM-ANOVA also corroborated that there is a main effect of the proposed target audience on the ratings by both trainees and attendings.

**Conclusions:**

A gestalt-based rating system is not sufficiently reliable when recommending online educational resources to trainees and attendings. Trainees’ gestalt ratings for recommending resources for both groups were especially unreliable. Our findings suggest the need for structured rating systems to rate online educational resources.

**Electronic supplementary material:**

The online version of this article (doi: 10.1007/s40037-017-0343-3) contains supplementary material, which is available to authorized users.

## What this paper adds

Online educational resources are increasing in numbers and popularity. As medical education shifts towards a flipped classroom model, more and more trainees and attendings are beginning to use these resources. We showed that both attendings and trainees are unable to make sufficiently reliable or consistent gestalt recommendation of online educational resources between key stakeholder groups. Differences in rater reliability may be due to differences in inferences made about the target audience, use of different reference standards, and lack of clarity in how to convert gestalt opinion into numerical rating. Future investigations should look at existing quality indicators identified for secondary educational resources to devise a structured rating system to consistently and reliably rate online educational resources.

## Introduction

Disruptive technology is changing medical education, particularly through the proliferation of blogs and podcasts [1–3]. Medical educators can easily produce and distribute educational materials, shifting control of curricular content away from textbooks into the hands of teachers and, increasingly, learners themselves. Similarly, the rising popularity of online educational resources alters educators’ monopoly on knowledge and creates opportunities for asynchronous learning, both integrated into formal curricula (co-curricular) or for independent, self-directed learning (extra- or co-curricular learning) [2, 4, 5].

Critics of online educational resources often point to their lack of formal quality control as a danger resulting from their ease of dissemination [6]. As outlined in Christensen’s model of disruptive innovation, this is a common thread with many disruptive products [7]. Disruptive innovations gain a foothold as simple, convenient, and inexpensive low-quality alternatives, which then quickly begin improving in quality. Historically, textbooks and lectures were accepted as being valuable without their quality being formally scrutinized; they benefited from being the only products available. Despite being largely unproven, to gain mainstream acceptance and disrupt the established order, the digital parallels of textbooks (blogs) and lectures (podcasts) [8] will have to provide quality assurance that rivals or surpasses these traditional media.

The widespread availability of online educational resources presents challenges to both educators and trainees. These secondary resources are available to trainees of all levels, despite their varying levels of expertise and needs. For example, junior trainees may require more elementary resources and assistance identifying their areas of need. Additionally, trainees of different levels rank domains of quality differently, for example, residents value ‘entertainment’ in podcasts more than attendings [2]. There is a perception that attending level staff would be better able to evaluate the quality of online educational resources than their trainees; however, the ability of clinicians of any level to evaluate these resources only begun to be assessed.

The increasing popularity of online educational resources suggests that free, independently-produced online medical education content is not simply a passing novelty. Trainees of all levels will continue to formally and informally recommend online educational resources that are produced and disseminated in very different ways to peers, near-peers, and supervisors. There are ample systems for scaffolding critical appraisal of primary literature (e. g. the JAMA User’s Guide [9], Best Evidence Medical Education [BEME] global scale [10], modified Newcastle–Ottawa Scale [m-NOS] [11], Medical Education Research Study Quality Instrument (MERSQI) [12]) and for determining literature relevance for front-line practitioners [13, 14]. However, a recent systematic review by Paterson et al. (2015) showed that very little has been specifically written about how to best evaluate secondary online medical resources [15]. This prompted the reporting of a wide range of themes in more broad educational literature around quality assessment of online educational resources [15]. Their published list included 151 quality indicators, under three main subthemes (Credibility, Content, and Design), which was deemed too unwieldy and practically difficult for use by individuals attempting to rate the quality of a single blog post [15]. Subsequent work included two Delphi studies which distilled this exhaustive list to something shorter [16, 17] and a study which derived two critical appraisal tools (METRIQ-5 and METRIQ-8) from the Delphi data [18]. While these efforts are ongoing given the success of initiatives such as the BEME global scale [10] and the Best Evidence in Emergency Medicine (BEEM) relevance scale [13, 14] (which uses a simple gestalt rating scale to capture the reader’s general impression regarding a journal publication), suggests that a similarly simple gestalt method might capture a reliable assessment of a single online educational resource.

In this study, we had two aims. The first was to assess whether attending educators and trainees are able to recommend online educational resources reliably using a gestalt-based rating system; the second was to determine how these groups’ impressions of these posts are correlated.

## Methods

### Study design

In this online survey-based study, participants were asked to read and assess 40 pre-selected blog posts. These 40 blog posts were divided into four separate survey blocks to avoid survey fatigue. Google Surveys (Google, Mountainview, CA, USA) were used to collect the data. A modified Dillman technique [19] optimized survey response rates by incorporating three reminder emails. This study was granted exemption by the Hamilton Integrated Research Ethics Board chairperson as it did not pertain to human subjects research and was deemed be part of a quality assurance/programme development initiative.

### Selection of participants and blog posts

A modified snowball technique [20] identified 54 participants (33 trainees, including both medical students and residents, and 21 attending physicians in emergency medicine) from North America. Each collaborator identified and nominated faculty educators, residents, and medical students associated with one of 22 institutions. The 40 featured blog posts (Appendix A, which may be downloaded at this web address: http://metriqstudy.org/wp-content/uploads/2016/03/Appendix-A.pdf) were randomly selected from those which had previously been reviewed by the Academic Life in Emergency Education Approved Instructional Resources (ALiEM AIR) series [21] as being high quality educational posts. Only participants who completed the ratings were included in this study for the purposes of the reliability and correlational analyses.

### Outcome measures

Demographic information was collected at the beginning of the survey, including: gender, country of residence, medical school attended, year of graduation, affiliated academic institution, level of training (medical student, resident, or attending), current institutional rank for attending physicians, number of years in practice, advanced degrees, involvement with educational resources (blogs, podcasts, and/or other educational materials development), and educational administrative/leadership roles.

Participants read and assessed each of the 40 blog posts using two seven-point gestalt-based rating scales. The first scale asked: ‘Would you recommend this resource to a learner?’ The second scale asked: ‘Would you recommend this resource to an attending physician for continuing medical education?’ Both gestalt rating scales were anchored at 1 by the statement, ‘no, this is an inappropriate resource for this audience,’ and 7 by the statement, ‘yes, this is a great resource for this audience’. Participants who were uncertain could indicate that they were ‘unsure’. This scale was piloted with a group of residents (*n* = 3) and attendings (*n* = 4) prior to study implementation to determine if it assisted them to quantify their gestalt impression of a post.

### Analysis

Data collected using Google Surveys were analyzed using SPSS (version 23, IBM Corporation, Redmond, WA). We used the following method to describe the relationship between the raters (i. e. trainees vs. attendings who are *making *the recommendations) and the gestalt rating scale used (i. e. gestalt recommendations *targeted to* a trainee audience vs. gestalt recommendations *targeted to* an attending audience).

#### Part 1: Consistency within the groups

Single measures, two-way random effects model (since we treated the trainee and attending raters as random samplings of the two populations) intraclass correlation coefficients (ICC) were calculated for each of the four rating groups: trainee recommended post for trainee, trainee recommended post for attending, attending recommended post for trainee, and attending recommended post for attending. The ICC is a measure of reliability. For our study, we calculated ICCs to assess agreement within a group of raters along the same rating scale.

#### Part 2: Correlations between group mean ratings

The mean scores for each rating group were calculated with standard deviations (SD). A Pearson correlation was used to compare the differences between groups’ opinions on a given blog post, to show if there was consistency between groups (reliability of ratings across both raters and targets). Family-wise adjustments were made for multiple comparisons using the Bonferroni technique. As we planned to complete four hypothetically driven comparisons in the Pearson correlation; the significant *p*-value was therefore set at 0.01 and below.

#### Part 3: Repeated measures ANOVA

When comparing large numbers of raters, it is important to analyze the data in a non-aggregated fashion [22]. We used a repeated measures analysis of variance (RM-ANOVA) to compare the effect of the different rating scales (gestalt recommendation targeted to trainees vs. gestalt recommendation targeted to attendings) on the blog ratings. We also stratified the data by level of training (i. e. trainee and attending) as a between-subjects variable, and utilized the blog ratings across the two scales to run the RM-ANOVA. This analysis required a data imputation technique since there were sufficient missing data points (i. e. ‘unsure’) to run the RM-ANOVA analysis. As such, we substituted the grand mean rating (across both scales) for this missing data as described in the literature [23].

## Results

A total of 54 volunteer collaborators were recruited for this rating exercise. Table 1 depicts their demographics. The mean gestalt score and standard deviation (SD) for each blog post (are available in the Online Supplementary Data).

**Table 1:** Demographics of our collaborators who participated in rating the online educational resources

	Trainee raters (*n* = 33)		Faculty raters (*n* = 21)	
Country of origin	9.1% United States of America		70.8% United States of America	
	90.9% Canada		23.8% Canada	
Years in practice at the time of enrollment	0 years in practice (All are trainees)		9.6 years in practice (SD 9.9)	
Academic affiliations	Year 1 medical students	27.2%	Full professor	9.5%
	Year 2 medical students	45.5%	Associate professor	19.0%
	Year 3 medical students	6.1%	Assistant professor	61.9%
	Year 4 medical students	0%	None	9.5%
	First year residents	0%		
	Second year residents	3.0%		
	Third year residents	6.1%		
	Fourth year residents	9.1%		
	Fifth year residents	3.0%		

*SD* standard deviation

### Consistency of ratings within groups

Our results showed that there was poor-to-fair agreement amongst both trainees and attendings using the gestalt-aligned 7‑point scale (Table 2). Traditionally, ICC measures of 0.1–0.2 are considered poor, 0.3–0.4 are considered fair, 0.5–0.6 considered moderate, 0.7–0.8 indicates strong agreement, and >0.8 indicates almost perfect [24].

**Table 2:** Reliability of recommendations by trainees and attending physicians

	Single measure ICC for recommendation for trainees to use resource for learning (95% CI)	Single measure ICC recommendation for attendings to use resource for learning (95% CI)
Trainee raters	0.21 (0.13–0.39) *p* < 0.001	0.16 (0.09–0.30) *p* < 0.001
Attending raters	0.27 (0.18–0.41) *p* < 0.001	0.22 (0.14–0.35) *p* < 0.001

*ICC* intra-class correlation coefficient

### Correlations between group mean ratings

When comparing rater behaviour, we found a moderate association between the trainees’ mean ratings of the resources for other trainees and trainees’ mean ratings of the resources for attendings (Pearson’s r = 0.56, *p* < 0.001). Attendings showed stronger correlation between scores for various blog posts, regardless of their target audience (Pearson’s r = 0.74, *p* < 0.001).

When considering the target population, trainees and attendings were both moderately consistent in their recommendations. Both rater populations had a moderate correlation for posts when they were asked to consider trainees as the target audience (Pearson’s r = 0.72, *p* < 0.001) and when considering attendings as the target audience (Pearson’s r = 0.61, *p* < 0.001). Each of the correlations was significant even after a Bonferroni correction was made to set the significance at *p* = 0.01.

### RM-ANOVA findings

There was no difference between the two rater populations (trainees and attendings) and their ratings of the online educational resources within each scale (F(1.47) = 1.24, *p* = 0.27). However, the results of the RM-ANOVA showed that there was a main effect (F(1.47) = 15.2, *p* < 0.01) of the different gestalt rating scales on the ratings of each group, suggesting that each scale prompted raters to rate the blog posts differently.

We also detected an interaction between the level of training (i. e. trainee vs. attending) on the effect of the gestalt rating scale (F(1.47) = 10.5, *p* < 0.01), meaning the level of training for an individual tended to affect the use of the scale. Taking this finding into consideration with the high Pearson correlation between the mean ratings of attendings regardless of the target audience and the slightly higher ICCs attendings’ gestalt ratings for both groups, this main effect may suggest that attendings are using the scale slightly more homogeneously. There was no interaction detected between the gestalt rating scale used and the blog post (F(1.47) = 3.84, *p* = 0.06) nor between the level of training, gestalt rating scale, and blog post (F(1.47) = 0.43, *p* = 0.52).

## Discussion

The dissemination of online educational resources is often through informal recommendation channels, such as word of mouth and social media. Our study findings suggest that we may need more consistent methods to evaluate and recommend these resources because gestalt ratings are not as consistent as we had previously hoped. With the increasing numbers of available online educational resources and a shift to a flipped classroom model, online resources could have substantial utility in providing didactic learning, thereby allowing engaged discussions to be held in the classroom [25, 26]. However, when trainees are evaluating resources for other trainees (ICC = 0.21 (0.13–0.39)) and attendings (ICC = 0.16 (0.09–0.30)), their recommendations are unreliable. Furthermore, attending educators may be unable to provide reliable recommendations for trainees (ICC (95% CI) = 0.27 (0.18–0.41)).

### Effect of the target audience

When recommending resources for the same audience, both rater pools had moderately correlated scores (trainee and attending recommended post for trainee, Pearson’s r = 0.72, *p* < 0.001; trainee and attending recommended post for attending, Pearson’s r = 0.61, *p* < 0.001) suggesting that the raters identified some common characteristics, which steered them towards rating certain blogs similarly. These findings are corroborated by our RM-ANOVA, which found a main effect of the gestalt rating scale (i. e. ‘would you recommend this resource to a learner?’ vs. ‘would you recommend this to an attending?’). Thus, when considering a similar audience, both rater groups of made similar inferences about the needs and quality indicators for the intended audience.

Raters likely make similar inferences about the needs of the intended target audiences (e. g. expected level of medical knowledge, need for the resource, and assumed quality markers). Several factors may have contributed to the large variability in ratings, resulting in lower ICCs. For example, different inferences about the target demographic among raters may have contributed to variability [27]. Also, with the lack of clear criteria and limited understanding of each number on the 7‑point scale, raters may have been unable to have a clear and consistent strategy when converting their judgments about a resource into a numerical rating [28].

### Attending rating for trainees and attending colleagues

One of the interesting findings in our study was that the RM-ANOVA detected an interaction between the level of training (e. g. trainee vs. attending) on the effect of the scale (F(1.47) = 10.5, *p* < 0.01). This suggested that the attending raters likely use some common criteria to discern the quality of resources, regardless of the proposed intended audience.

Previous literature has shown there is consensus among programme directors regarding quality markers such as appropriate literature referencing in online educational resources [2]. There are likely other not-yet-discovered common quality markers that may connote quality to attending physicians. One recent study identified a list of 151 quality indicators that may be useful in assessing secondary online educational resources in medical education [15]. We propose that further research in this domain will be required to reduce this list and create a tool to guide trainees and/or attending educators towards good quality online educational resources.

### Future directions

The variability in numerical ratings using the 7‑point gestalt scale might have been improved through better calibration by developing a shared mental model amongst raters. For example, Kogan and colleagues identified multiple factors affecting faculty members’ ratings of residents during training encounters [27] and similar effects were likely present within our rater pools. For instance, our raters might have had frame of reference problems with individual raters who have different levels of clinical expertise, preferred practices, and medical knowledge, affecting their judgments about the various resources. Moreover, our gestalt scales did not have explicit criteria that raters could use to examine the blog posts, leading to an inordinately high level of inference. Future research may find simple interventions, e. g. anchors at each point on the rating scale, which may improve scoring. Finally, as with all number-based scales, raters may have lacked a strategy to convert clinical judgment into a numerical rating.

Our findings suggest that tools are needed to provide more accurate and reliable assessments. There are many scores available to assess primary literature and systematic reviews, such as *Preferred Reporting Items for Systematic Reviews and Meta-Analyses *(PRISMA) [29, 30] and Cochrane Collaborative [31], but none exists to assess secondary educational resources such as blogs. The only score similar to our gestalt-based rating system is the *Best Evidence in Emergency Medicine (BEEM)* rating scale [13], which attempts to recommend only practice-changing studies to emergency physicians and requires a relatively small number of raters to do so (12–15 raters) [13]. To overcome the problems detected in our study regarding rater consistency, we suggest that the development of a shared mental model to help identify and evaluate the quality of online educational resources, similar to the way in which evidence-based medicine guides (e. g. *JAMA* User’s Guide [9], PRISMA [29, 30], or BEEM rating scale [13]) help readers to consider similar aspects of a study when critically appraising primary literature.

### Limitations

The generalizability of our results is limited by our sample size and sampling procedure. Due to a limited number of trainees, we lacked the power to be able to distinguish between medical students and residents as separate subgroups in this study. The varying levels of knowledge in this diverse demographic group may have skewed the recommendations as medical students may have difficulty discerning accuracy and relevance of clinical material [2, 3]. Given that our study used a snowball sampling technique (rather than a random sampling technique), our findings may not be generalizable. As such, the confidence intervals and *p*-values within our paper should be interpreted with this in mind.

Some attending physicians participating in this study had affiliations with blog sites, which may have biased their ratings. However, the inconsistency of ratings in our study suggested that attendings who themselves have written or contributed to online educational resources were also having difficulty making consistent recommendations. This highlights that unfamiliarity with rating secondary resources is likely to be a major problem in recommending online educational resources [17].

The use of parametric tests (e. g. Pearson’s correlation, ANOVA, or ICC) on our small dataset could be criticized. However, previous methodological leaders in our field have argued convincingly that they provide robust analysis even in small populations [32].

The bulk of our missing data was generated due to uncertainty with ratings about specific items. To complete the RM-ANOVA, we chose to use the most conservative approach to account for missing data. We substituted the grand mean (i. e. the mean score of all ratings generated by either gestalt scale) when raters were uncertain of how to use the survey rating or there was another cause for missing data (i. e. incomplete ratings) so as to minimize the effect of these missing ratings on the analysis. Substituting the grand mean during the RM-ANOVA effectively nullifies the effect of these ratings on the analysis.

Lastly, our results were confounded by the use of 7‑point scales anchored to the idea of recommendation. There were no clear criteria or anchors defined to convert gestalt opinion into a numerical rating on a scale, which may have contributed to the inconsistency and variability in ratings [28]. While we intentionally kept our anchors vague so that we might best approximate a reader’s innate gestalt, it is possible that a more directed question (e. g. ‘Would you recommend this to a postgraduate year 1 resident?’) would yield a more reliable answer. However, if such a refinement in anchoring questions would have helped, we would have expected a much higher agreement than an ICC of 0.22 in the attending recommendation to their attending-peers.

## Conclusion

Gestalt recommendation of online educational resources by trainees and attendings may not be sufficiently reliable or consistent between these key stakeholder groups. This may be due to a difference in inferences made about the target audience, use of different reference standards, and a lack of clarity in how to convert gestalt opinion into a numerical rating. However, our study provides evidence that there are some common, but as yet unidentified, criteria used by both trainees and attendings to discern the quality of a resource when making recommendations. We propose that future investigations focus on tailoring the existing list of quality indicators in secondary educational resources in order to devise a scale that will allow greater consistency and reliability in future trainee and attending recommendations of online educational resources.

## Caption Electronic Supplementary Material

Supplementary Table: Mean gestalt rating scores for blog posts by parent website

## References

[1] Cadogan M, Thoma B, Chan TM, Lin M (2014). Free Open Access Meducation (FOAM): the rise of emergency medicine and critical care blogs and podcasts (2002–2013). Emerg Med J.

[2] Purdy E, Thoma B, Bednarczyk J, Migneault D, Sherbino J (2015). The use of free online educational resources by Canadian emergency medicine residents and program directors. CJEM.

[3] Mallin M, Schlein S, Doctor S, Stroud S, Dawson M, Fix M (2014). A survey of the current utilization of asynchronous education among emergency medicine residents in the United States. Acad Med.

[4] Nickson CP, Cadogan MD (2014). Free Open Access Medical education (FOAM) for the emergency physician. Emerg Med Australas.

[5] Pearson D, Bond M, Kegg J (2015). Evaluation of social media use by emergency medicine residents and faculty. West J Emerg Med.

[6] Brabazon T (2006). The Google Effect: googling, blogging, wikis and the flattening of expertise. Libri.

[7] Christensen C, Raynor ME (2003). The Innovator’s Solution: creating and sustaining successful growth.

[8] Thoma B, Chan T, Benitez J, Lin M (2014). Educational scholarship in the digital age: a scoping review and analysis of scholarly products. Winnower.

[9] Guyatt G, Meade MO, Rennie D, Cook DJ (2015). User’s guides to the medical literature: a manual for evidence-based clinical practice.

[10] Littlewood S, Ypinazar V, Margolis SA (2005). Early practical experience and the social responsiveness of clinical education: systematic learning in practice early practical experience and the social responsiveness of clinical education: systematic review. BMJ.

[11] Wells G, Shea B, O’Connell D, et al. The Newcastle-Ottawa Scale (NOS) for assessing the quality of nonrandomised studies in meta-analyses [Internet]. Available from: http://www.ohri.ca/programs/clinical_epidemiology/oxford.asp. Accessed Feb. 2, 2017.

[12] Cook DA, Levinson AJ, Garside S (2011). Method and reporting quality in health professions education research: a systematic review. Med Educ.

[13] Worster A, Kulasegaram K, Carpenter CR (2011). Consensus conference follow-up: inter-rater reliability assessment of the Best Evidence in Emergency Medicine (BEEM) rater scale, a medical literature rating tool for emergency physicians. Acad Emerg Med.

[14] Carpenter CR, Sarli CC, Fowler S (2013). Best Evidence in Emergency Medicine (BEEM) rater scores correlate with publications’ future citations. Acad Emerg Med.

[15] Paterson QS, Thoma B, Milne WK, Lin M, Chan TM (2015). A systematic review and qualitative analysis to determine quality indicators for health professions education blogs and podcasts. J Grad Med Educ.

[16] Thoma B, Chan TM, Paterson QS, Milne WK, Sanders JL, Lin M (2015). Emergency medicine and critical care blogs and podcasts: establishing an international consensus on quality. Ann Emerg Med.

[17] Lin M, Thoma B, Trueger NS, Ankel F, Sherbino J, Chan T (2015). Quality indicators for blogs and podcasts used in medical education: modified Delphi consensus recommendations by an international cohort of health professions educators. Postgrad Med J.

[18] Chan TM, Thoma B, Krishnan K, Lin M, Carpenter C, Astin M (2016). Derivation of two critical appraisal scores for trainees to evaluate online educational resources: A METRIQ Study. West J Emerg Med.

[19] Dilman DA (2000). Mail and Internet surveys: the tailored design method.

[20] Heckathorn D (2011). Sampling, Snowball versus respondent-driven. Sociol Methodol.

[21] Lin M, Joshi N, Grock A, Swaminathan A, Morley EJ, Branzetti J (2016). Approved Instructional Resources (AIR) Series: a national initiative to identify quality emergency medicine blog and podcast content for resident education. J Grad Med Educ.

[22] Leppink J, van Merriënboer JJG (2015). The beast of aggregating cognitive load measures in technology-based. Learning.

[23] Graham JW (2012). Missing data: Analyis and design.

[24] Norman GR, Streiner DL (2008). Health Measurement Scales: A practice guide to their development and use. Third.

[25] Mehta NB, Hull AL, Young JB, Stoller JK (2013). Just Imagine. Acad Med.

[26] Prober CG, Khan S (2013). Medical Education Reimagined. Acad Med.

[27] Kogan JR, Conforti L, Bernabeo E, Iobst W, Holmboe E (2011). Opening the black box of clinical skills assessment via observation: A conceptual model. Med Educ.

[28] Hanson JL, Rosenberg A, Lane JL (2013). Narrative descriptions should replace grades and numerical ratings for clinical performance in medical education in the United States. Front Psychol.

[29] Moher D, Liberati A, Tetzlaff J, Altman DG, Grp P (2009). Preferred reporting items for systematic reviews and meta-analyses: the PRISMA statement (reprinted from annals of internal medicine). Phys Ther.

[30] Moher D, Liberati A, Tetzlaff J, Altman DG (2009). Preferred reporting items for systematic reviews and meta-analyses. Ann Intern Med.

[31] Higgins JP, Green S (2008). Cochrane Handbook for Systematic Reviews of Interventions: Cochrane Book Series.

[32] Norman G (2010). Likert scales, levels of measurement and the ‘laws’ of statistics. Adv Health Sci Educ Theory Pract.

