# Supplementary material for: Gestalt assessment of online educational resources may not be sufficiently reliable and consistent
Source: Perspect Med Educ. 2017 Feb 27;6(2):91–8. doi: 10.1007/s40037-017-0343-3 (PMC5383576; doi:10.1007/s40037-017-0343-3)
Supplement: Supplementary file 1 — Supplementary Table: Mean gestalt rating scores for blog posts by parent website [file 40037_2017_343_MOESM1_ESM.docx]

**Online supplementary data**

Mean gestalt rating scores for blog posts by parent website

| Blog posts organized by parent website^†^ | Median trainee gestalt recommendation score for trainees (SD) | Median trainee gestalt recommendation score for attendings (SD) | Median trainee gestalt recommendation score for trainees (SD) | Median trainee gestalt recommendation score for attendings (SD) | |
| --- | --- | --- | --- | --- | --- |
| *EM LYCEUM* |  |  |  |  | |
| Eye emergencies, ‘answers’ | 4.82 (1.42) | 5.33 (1.43) | 5.62 (1.50) | 5.95 (1.12) | |
| Epistaxis, ‘answers’ | 5.39 (1.32) | 5.36 (1.37) | 5.86 (0.96) | 5.43 (1.08) | |
| Abscess, ‘answers’ | 4.50 (1.42) | 5.26 (1.43) | 5.48 (1.81) | 5.71 (1.38) | |
| *The Poison Review* |  |  |  |  | |
| Review of scorpion envenomation | 3.85 (1.56) | 3.67 (1.51) | 4.48 (1.33) | 4.00 (1.84) | |
| Make the diagnosis, Sherlock! | 4.55 (1.46) | 4.38 (1.68) | 4.14 (1.49) | 3.62 (1.80) | |
| *Academic Life in Emergency Medicine (ALiEM)* |  |  |  |  | |
| Are acetaminophen levels necessary in all overdose patients? | 5.76 (1.37) | 5.52 (1.64) | 5.76 (1.26) | 5.05 (1.56) | |
| Approach to difficult vascular access | 5.67 (1.55) | 5.91 (1.35) | 6.05 (1.07) | 4.62 (1.86) | |
| Antidiabetic medications: hypoglycaemic potential in overdose | 5.37 (1.45) | 5.01 (1.87) | 5.01 (1.36) | 4.29 (1.65) | |
| PV card: local anaesthetic toxicity calculations | 4.70 (1.35) | 5.52 (1.16) | 5.05 (1.28) | 4.76 (1.22) | |
| Carbon monoxide poisoning: common questions and dilemmas | 5.74 (1.10) | 5.56 (1.19) | 5.71 (1.06 | 4.90 (1.55) | |
| PV card: diagnosis of DVT (ACCP guidelines) | 5.85 (1.41) | 5.89 (1.19) | 5.35 (1.27) | 4.20 (1.64) | |
| Thyroid storm: treatment strategies | 5.70 (1.32) | 5.93 (1.07) | 6.20 (0.70) | 5.30 (1.22) | |
| Trick of the trade: Use the angiocatheter for central lines | 4.22 (1.34) | 5.38 (1.20) | 4.78 (1.40) | 3.83 (1.72) | |
| 5 Tips in managing acute salicylate poisoning | 5.91 (1.02) | 5.68 (1.36) | 6.17 (0.71) | 5.44 (1.10) | |
| Trick of the Trade: The PIPP for deep peripheral IVs in obese patients | 4.22 (1.99) | 5.44 (1.28) | 4.7 (1.17) | 4.60 (1.35) | |
| Diagnosing hyperthyroidism: answers to 7 common questions | 6.48 (0.70) | 5.73 (1.43) | 5.25 (1.07) | 4.42 (1.46) | |
| DVT: Wells criteria and D-dimers happy together | 5.33 (1.33) | 5.00 (1.36) | 5.20 (1.15) | 5.00 (1.45) | |
| Paucis verbis: Neutropenic fever in cancer patients | 5.54 (1.58) | 5.79 (1.06) | 5.85 (0.99) | 5.00 (1.65) | |
| *Emergency medicine Ireland* |  |  |  |  | |
| Methadone in the emergency room | 4.76 (1.50) | 4.36 (1.65) | 4.47 (1.31) | 3.68 (1.53) | |
| *Boring EM* |  |  |  |  | |
| Boring question: Does this medication cause long QT? | 5.76 (1.20) | 4.97 (1.38) | 5.25 (1.07) | 4.25 (1.52) | |
| *The Skeptics’ Guide to EM* |  |  |  |  | |
| SGEM #31: She’s got legs (DVT and thrombophlebitis) | 5.12 (1.43) | 4.91 (1.69) | 5.10 (1.22) | 4.86 (1.42) | |
| *Life in the Fastlane* |  |  |  |  | |
| Half an 8 ball | 6.06 (1.17) | 5.55 (1.20) | 6.33 (0.91) | 5.33 (1.28) | |
| The Red Eye Challenge | 5.81 (1.33) | 5.15 (1.43) | 5.80 (0.95) | 4.75 (1.65) | |
| *Paediatric EM Morsels* | 5.81 (1.33) | 5.15 (1.43) | 5.80 (0.95) | 4.75 (1.65) | |
| Wet purpura and ITP | 5.58 (1.10) | 4.46 (1.75) | 5.68 (1.16) | 4.89 (1.91) | |
| Cerebral oedema and diabetic ketoacidosis | 5.58 (1.33) | 5.69 (1.23) | 5.72 (1.18) | 4.72 (1.74) | |
| Penicillin for pneumonia | 5.58 (1.30) | 5.12 (1.70) | 5.18 (1.07) | 4.65 (1.46) | |
| Recurrent croup | 5.73 (0.96) | 5.04 (1.17) | 5.67 (1.19) | 4.67 (1.75) | |
| *EM Basic* |  |  |  |  | |
| EM basic: acetaminophen overdose | 5.52 (1.76) | 4.52 (1.87) | 5.00 (1.45) | 3.57 (1.66) | |
| *The Original Kings of County* |  |  |  |  | |
| Why are we repeating an already negative lower extremity Doppler? | 4.56 (1.25) | 5.00 (1.39) | 5.14 (1.39) | 4.95 (1.47) | |
| *Don’t forget the bubbles* |  |  |  |  | |
| Diabetes insipidus | 6.44 (0.89) | 4.78 (1.45) | 5.43 (1.16) | 4.19 (1.33) | |
| Peri-orbital V orbital cellulitis | 5.22 (1.22) | 4.42 (1.53) | 5.50 (1.15) | 3.80 (1.44) | |
| *EMCrit* |  |  |  |  | |
| Podcast 98: cyclic (tricyclic) antidepressant overdose | 4.30 (1.56) | 4.93 (1.41) | 5.57 (1.43) | 5.48 (1.44) | |
| *ERCast* |  |  |  |  | |
| Art of chemical takedown | 4.42 (1.68) | 5.38 (1.53) | 4.76 (1.87) | 5.47 (1.91) | |
| Rivaroxaban (Xarelto) for DVT | 4.70 (1.38) | 6.04 (0.98) | 5.9 (1.02) | 6.20 (0.89) | |
| TRAAPPED SILO SAFE Mnemonic | 5.74 (1.38) | 4.81 (1.52) | 4.95 (1.57) | 4.30 (1.84) | |
| *EM literature of note* |  |  |  |  | |
| Droperidol never killed anyone | 2.85 (1.59) | 3.23 (1.63) | 3.50 (1.32) | 3.15 (2.13) | |
| R.E.B.E.L. EM |  |  |  |  | |
| Is there any benefit to an initial insulin bolus in diabetic ketoacidosis? | 5.19 (1.41) | 5.88 (1.14) | 5.47 (1.12) | 4.47 (1.61) | |
| HQMedEd |  |  |  |  | |
| Trial of ketamine vs. haloperidol for severe prehospital agitation | 5.11 (1.40) | 4.67 (1.52) | 5.70 (0.98) | 5.30 (1.13) | |
| Dr. Smith’s ECG Blog |  |  |  |  | |
| Agitation and tachycardia | 5.11 (1.40) | 4.67 (1.52) | 5.70 (0.98) | 5.30 (1.13) | |
| TRAAPPED SILO SAFE Mnemonic |  |  |  |  | |
| Antibiotics for clinically diagnosed acute sinusitis in adults | 4.07 (1.52) | 4.96 (1.51) | 5.15 (1.09) | 5.00 (1.30) | |
|  | | | | |  |

^†^Refer to http://metriqstudy.org/wp-content/uploads/2016/03/Appendix-A.pdf for hyperlinks to each blog post
